# *Anopheles plumbeus* (Diptera: Culicidae) in Europe: a mere nuisance mosquito or potential malaria vector?

**DOI:** 10.1186/1475-2875-11-393

**Published:** 2012-11-26

**Authors:** Francis Schaffner, Isabelle Thiéry, Christian Kaufmann, Agnès Zettor, Christian Lengeler, Alexander Mathis, Catherine Bourgouin

**Affiliations:** 1 Vector Entomology Unit, Institute of Parasitology, Vetsuisse Faculty, University of Zürich, Winterthurerstrasse 266a, Zürich, CH-8057, Switzerland; 2 Centre de Production et d’Infection des Anophèles, Plate-forme CEPIA, Institut Pasteur, 28 rue du Dr Roux, Paris, 75724, France; 3 Swiss Tropical and Public Health Institute, Socinstrasse 57, Basel, CH-4002, Switzerland; 4 Present address: Genetics and Genomics of Insect Vectors, Institut Pasteur, 28 rue du Dr Roux, Paris, 75724, France

## Abstract

**Background:**

*Anopheles plumbeus* has been recognized as a minor vector for human malaria in Europe since the beginning of the 20^th^ century. In recent years this tree hole breeding mosquito species appears to have exploited novel breeding sites, including large and organically rich man-made containers, with consequently larger mosquito populations in close vicinity to humans. This lead to investigate whether current populations of *An. plumbeus* would be able to efficiently transmit *Plasmodium falciparum*, the parasite responsible for the most deadly form of malaria.

**Methods:**

*Anopheles plumbeus* immatures were collected from a liquid manure pit in Switzerland and transferred as adults to the CEPIA (Institut Pasteur, France) where they were fed on *P. falciparum* gametocytes produced *in vitro*. *Anopheles gambiae* mosquitoes served as controls. Development of *P. falciparum* in both mosquito species was followed by microscopical detection of oocysts on mosquito midguts and by sporozoite detection in the head/thorax by PCR and microscopy.

**Results:**

A total of 293 wild *An. plumbeus* females from four independent collections successfully fed through a membrane on blood containing *P. falciparum* gametocytes. Oocysts were observed in mosquito midguts and *P. falciparum* DNA was detected in head-thorax samples in all four experiments, demonstrating, on a large mosquito sample, that *An. plumbeus* is indeed receptive to *P. falciparum* NF54 and able to produce sporozoites. Importantly, the proportion of sporozoites-infected *An. plumbeus* was almost similar to that of *An. gambiae* (31 to 88% *An. plumbeus versus* 67 to 97% *An. gambiae).* However, the number of sporozoites produced was significantly lower in infected *An. plumbeus*.

**Conclusion:**

The results show that a sample of field-caught *An. plumbeus* has a moderate to high receptivity towards *P. falciparum*. Considering the increased mobility of humans between Europe and malaria endemic countries and changes in environment and climate, these data strongly suggest that *An. plumbeus* could act as a vector for malaria and thus significantly contribute to increasing the malaria transmission risk in Central-Western Europe*.* In locations showing high vulnerability to the presence of gametocyte carriers, the risk of transmission of malaria by *An. plumbeus* should be considered.

## Background

Global changes in health of humans and livestock are of concern to policy and decision making bodies. Over the last fifteen years, autochthonous cases and epidemic outbreaks of vector-borne diseases have occurred in Europe (e.g. bluetongue [1], chikungunya [2], dengue [3,4], Schmallenberg disease [5], Usutu virus infection [6]), including local transmission of malaria in France, Germany, Greece, Italy, and Spain [7-11]. Among the factors that contribute to the emergence or re-emergence of vector-borne diseases are the increased mobility of humans, livestock, and pathogens, as well as environmental modification, and climate change [12]. This emphasizes the need to evaluate or re-evaluate the capability of European Anopheline mosquitoes to allow local transmission of malaria, in particular of *Plasmodium falciparum,* which is responsible for the most deadly form of the disease.

Several factors have contributed to the decline and disappearance of malaria in Western Europe and most of the Mediterranean countries during the 19^th^ and early 20^th^ centuries [8,13], including improvements in socio-economic conditions (particularly the separation of human and animal housing), the development and widespread use of efficient anti-malarial drugs, large-scale elimination of mosquito breeding sites through drainage, and improvement in mosquito control activities. Several *Anopheles* species had contributed to the transmission of malaria parasites in Europe, with *Plasmodium vivax* presumably being the prevalent species, although this is not fully ascertained [13]. The primary vector species belong to the Maculipennis complex with *Anopheles atroparvus* acting as the main vector in western, northern and central Europe, and *Anopheles labranchiae* and *Anopheles sacharovi* in southern regions. Other species have been considered as minor vectors, including further species of the Maculipennis complex (*Anopheles messeae*, *Anopheles maculipennis* s.s., *Anopheles melanoon*), as well as *Anopheles algeriensis*, *Anopheles claviger* s.s., *Anopheles cinereus*, *Anopheles hyrcanus*, *Anopheles plumbeus*, *Anopheles sergentii*, and *Anopheles superpictus*[14,15]. All the above mentioned species are still present in Europe [16,17]. Following malaria eradication campaigns, the abundance and distribution of the main vectors have been sustainably reduced in some areas but have recovered to initial levels in others [18,19]. The principal vectors of *P. vivax* (*An. atroparvus*, *An. labranchiae*, *An. sacharovi*) were refractory or had a low vector competence for *P. falciparum* in experimental investigations [20-22]. Recent studies of *An. labranchiae* populations collected in France and Italy confirmed that this species is receptive to *P. falciparum,* but exhibits a low vector competence [23,24].

**Figure 1:**
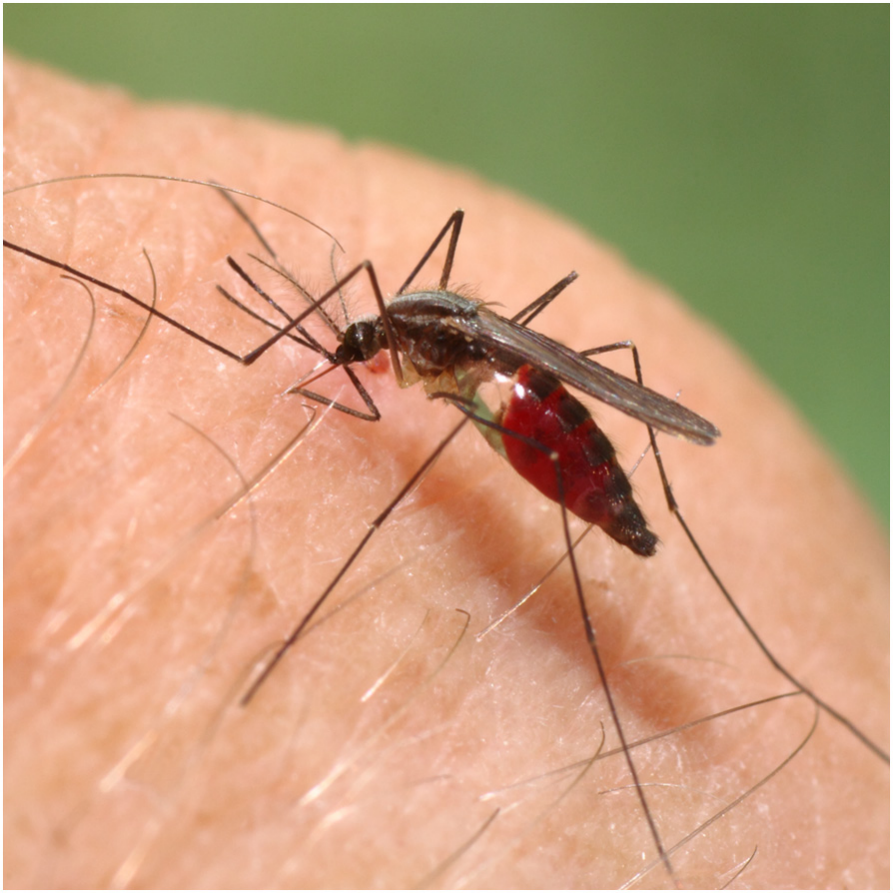
***Anopheles plumbeus *female (source: F. Schaffner/IPZ).**

*Anopheles plumbeus* (Figure 1) is widely distributed throughout Europe (with the exception of far-northern regions), the Middle East and North Africa [16,17]. The species has been the subject of recent attention due to increased abundance in human vicinity leading to strong nuisance as this mosquito is a fierce human biter (Schaffner, unpublished; [17,25]). This increase in abundance is likely the consequence of an expansion of larval habitats. Originally known as a dendrolimnic species, breeding almost exclusively in tree holes with correspondingly small adult populations due to the scarcity of such breeding sites, this mosquito has recently been shown to exploit a wider array of larval breeding sites such as septic tanks, catch basins, tires, cemetery vases, rain water casks [25-28]. These novel breeding sites are all man-made sites, usually rich in organic matter, and indeed, all cemetery vases colonized by *An. plumbeus* contained dead leaves (Schaffner, unpublished). Limited data are available with regard to this species’ competence for human malaria. *Anopheles plumbeus* was incriminated as a vector of *P. vivax*[29,30] and of *P. falciparum* in two cases of autochthonous malaria cases in Germany [7]. In 1920, Blacklock and Carter [31] experimentally infected one out of 11 *An. plumbeus* with *P. falciparum*. More recently, experimental feeding of *An. plumbeus* on blood containing *P. falciparum* gametocytes of the NF54 isolate led to the detection of oocysts in the midgut of three out of five mosquitoes [21] and of sporozoites in the salivary glands of six out of 10 [32].

This study shows that *An. plumbeus* mosquitoes from a field collection from Switzerland, with nearly three hundred females tested, are indeed competent towards *P. falciparum* NF54. The possibility of *An. plumbeus* becoming a more important vector of *P. falciparum* in Western Europe is discussed, considering the large number of malaria cases imported into the European Union every year (10,000 to 12,000 [33]) and the occurrences of episodes of higher than normal temperatures during summer [34,35] which may offer a more favourable environment for autochthonous transmission of *P. falciparum* in temperate parts of Europe.

## Methods

### Mosquitoes

*Anopheles plumbeus* immatures were collected from a liquid manure pit located in Bäretswil, canton of Zurich, in a hilly region of northern Switzerland. A high number of immatures were present in this large breeding site at all visits from May to November, 2010. Immatures were sampled on four occasions (June, July, September and November 2010), reared to the adult stage in the laboratory and maintained on a 10% glucose solution at room temperature (21°C ± 2°C) and 40% ± 10% relative humidity (RH) under a 14 h-10 h day-night photoperiod. From each collection, 200 to 250 *An. plumbeus* females were sent to the CEPIA (Institut Pasteur, Paris, France) 6 days prior to *P. falciparum* challenge. Upon arrival, females were maintained under standard conditions (25°C ± 1°C, 75% ± 5% RH and 12 h-12 h day-night photoperiod) and provided a 10% sucrose solution *ad libidum* before being starved from sugar 24 h prior feeding on blood containing *P. falciparum* gametocytes. As a control, the *An. gambiae* Ngousso strain, which is highly receptive to *P. falciparum*[36], was fed on the same gametocyte preparations. All *An. gambiae* females used as control were maintained on 10% sucrose supplemented with a 100 U penicillin-0.01% Streptomycin final concentration solution (Invitrogen, France) from adult emergence until the infection experiment to minimize bacterial flora and thus increase susceptibility to *P. falciparum* (Thiéry *et al.*, personal communication; [37]). This treatment was not applied to *An. plumbeus* in order to assess its receptivity under more natural conditions.

### Parasites and experimental feeding

*Plasmodium falciparum* isolate NF54 was cultured using a semi automated tipper table system [38], and subcultures were produced according to Mitri and colleagues [39] using fresh erythrocytes from anonymous blood donors (7% final haematocrit) provided by ICAReB platform, Institut Pasteur. Fourteen days after initiation of the subculture, parasitemia and gametocyte sex ratio were determined on Giemsa-stained smears, and gametocyte maturity was evaluated by checking for exflagellation of microgametes. Ten ml of 14 day-old gametocyte culture were centrifuged at 800 g for 5 min and the pellet was resuspended in a mixture of fresh red blood cells and AB human serum (EFS, Rungis, France) to a 40% final haematocrit. One ml of parasitized blood was poured in each blood feeder maintained at 37°C. The gametocyte density for the four experiments were 8.3, 4.2, 8.0 and 5.7 × 10^7^/ml, respectively. Mosquitoes were allowed to feed in the dark through a Parafilm® membrane for 15 min. Unengorged females were discarded while fully engorged females were transferred into small cages and provided with 10% sucrose containing 0.05% PABA (4-para-amino benzoic acid, Sigma, France) and 0.01% final concentration penicillin-streptomycin for *An. gambiae* only. To mimic *Anopheles* feeding behaviour in nature, additional uninfected fresh blood meals were provided to all mosquitoes 3 and 9 days post- *P. falciparum* ingestion (p.i.). Mosquitoes were kept at 25°C, 75% RH for the duration of the experiments.

### Analysis of infection

The presence of developing oocysts in mosquito midguts and of sporozoites in salivary glands was determined. Microscopic oocyst detection and quantification were performed on dissected midguts, stained with 0.1% mercuro-bromo fluorescein (Fluka, France) in phosphate buffered saline, at days 8 and 15 p.i. When available, at least 30 females per experiment and species were analysed. A female was considered infected if at least one oocyst was seen. At day 15 p.i., oocysts were recorded as either fully developed or ruptured. At this time point, the head-thorax tissue containing the salivary glands were isolated and stored at −20°C for PCR detection of sporozoite DNA as described [40]. In addition, quantification of sporozoites in head-thorax tissue was performed using the Ozaki method [41].

## Results and discussion

A total of 293 wild *An. plumbeus* females successfully fed through a membrane on blood containing *P. falciparum* gametocytes. Oocysts were observed at days 8 and 15 p.i. in mosquito midguts. In addition, *P. falciparum* DNA was detected in head-thorax samples in all four experiments. These results confirm, on a large mosquito sample, that *An. plumbeus* is receptive to *P. falciparum* NF54 infection and able to produce sporozoites (prevalence 31–88%). Detailed analysis of the results demonstrates that this European mosquito species has moderate to high receptivity to *P. falciparum*, compared to the CEPIA’s reference strain *An. gambiae* (Table 1). On day 15 after gametocyte ingestion, oocysts developed in 10 to 75% of *An. plumbeus* mosquitoes, while 67 to 100% *An. gambiae* harboured oocysts. At this time point, the overall mean number of oocysts developing in *An. plumbeus* (2–28.8) was lower than of *An. gambiae* (10.7–44.8). Comparing the infection prevalence at day 8 and day 15 reveals a slight decrease in *An. gambiae* while the opposite occurred in *An. plumbeus*. The decrease in *An. gambiae* prevalence could be due to the difficulty of detecting ruptured oocysts. The slight increase in *An. plumbeus* prevalence is suggestive of a slower development of *P. falciparum* in this species, with oocysts continuing their development between day 8 and day 15. However, in both species the mean number of oocysts decreased between day 8 and day 15, which, as mentioned above, is likely the result of the inability to detect oocysts that had ruptured. Therefore, the apparent discrepancy between increased prevalence of infection and decreased oocyst mean intensity at day 15 for *An. plumbeus* is suggestive of asynchrony of *P. falciparum* development in this mosquito species under this experimental setting. Importantly, the proportion of *An. plumbeus* harbouring sporozoites is high, ranging from 31 to 88%, not very much different from the proportion of sporozoite-positive *An. gambiae* (67 to 97%). This prevalence of sporozoite infection is higher than the prevalence of oocyst infection at days 8 or 15, which is not surprising considering the proportion of mosquitoes with few or undetectable oocysts and the sensitivity of PCR. The presence of sporozoites in *An. plumbeus* was then confirmed using the Ozaki method in the two experiments involving a large sample size and showing high oocyst prevalences (experiments 1 and 3, Table 1). The quantity of sporozoites was nevertheless lower than in *An. gambiae* when estimated per female harbouring at least one oocyst at day 15. This finding is consistent with the reduced oocyst load in *An. plumbeus.*

**Table 1:** ***Plasmodium falciparum *infection and dissemination in field-caught *An. plumbeus *and laboratory-reared *An. gambiae *Ngousso**

		***Anopheles plumbeus***				***Anopheles gambiae *Ngousso**			
	**Day of observation**^**a**^	**Exp. 1**	**Exp. 2**^**b**^	**Exp. 3**	**Exp. 4**	**Exp. 1**	**Exp. 2**	**Exp. 3**	**Exp. 4**
Prevalence (%) of midgut infection (no. positive / no. analysed)	8	67	nd^c^	40	4	100	100	87	73
		(20/30)		(12/30)	(1/24)	(30/30)	(30/30)	(26/30)	(22/30)
	15	75	15	43	10	100	100	67	70
		(36/48)	(2/13)	(22/51)	(7/70)	(32/32)	(24/24)	(20/30)	(21/30)
Intensity of infection: Mean no. of oocysts / positive female^d^ (range)	8	8.9 ± 6.3	nd^c^	50.4 ± 56.0	12.0 ± 0	42.4 ± 28.2	71.5 ± 63.3	29.5 ± 23.5	13.7 ± 13.6
		(2–25)		(1–171)	(12)	(3–122)	(1–233)	(1–74)	(1–48)
	15	5.3 ± 4.9	2.0 ± 0	28.8 ± 14.4	2.4 ± 2.1	22.4 ± 25.2	44.8 ± 36.4	26.4 ± 23.7	10.7 ± 10.1
		(1–18)	(2)	(4–63)	(1–6)	(1–112)	(1–146)	(1–79)	(1–45)
Prevalence (%) of females with sporozoites^e^ (no. positive / no. analysed)	15	83	31	88	57	97	88	67	70
		(40/48)	(4/13)	(45/51)	(40/70)	(31/32)	(21/24)	(20/30)	(21/30)
Intensity of infection: Mean no. sporozoites / positive female^f^ (no. analysed)	15	1,620	nd^c^	108	nd^c^	11,178	25,623	15,755	950
		(12)		(15)		(30)	(30)	(30)	(30)

Experiments 1 to 4 were performed in June, July, September and November, 2010, respectively.

^a^ After exposure to *P. falciparum* gametocytes.

^b^ Low number of *An. plumbeus* mosquitoes due to high mortality during transport.

^c^ Not determined.

^d^ Positive females are females with at least 1 detectable oocyst.

^e^ PCR detection [40] of *P. falciparum* DNA isolated from head/thorax.

^f^ Sporozoite quantification was performed using the Ozaki method [41]; total number was normalized to the oocyst prevalence at day 8.

*Anopheles plumbeus* has been described as a putative vector for *P. vivax* under central European [29,42] and Russian climate conditions [30]. Studies have found that it is a more efficient carrier of *P. vivax* than *An. atroparvus* and *An. claviger*[43]. The first experimental demonstration of *An. plumbeus* competence towards *P. vivax* and *P. falciparum* were reported nearly 100 years ago [31,44,45]. *Anopheles plumbeus* competence towards *P. vivax* has not been experimentally reevaluated since, mainly due to the difficulty of access to *P. vivax* gametocytes. In contrast, *An. plumbeus* ability to permit *P. falciparum* development to the sporozoite stage was recently shown under laboratory conditions on a sample of ten mosquitoes [32]. The present study demonstrates the development of *P. falciparum* sporozoites, the stage infective to humans, in a wild population of *An. plumbeus* and for a large number of specimens (n = 293) collected at different times during the yearly natural occurrence of the species. Although these results show that *An. plumbeus* developed less *P. falciparum* oocysts than *An. gambiae*, the proportion of mosquitoes harbouring sporozoites was similar in both species. Associated with its fierce human biting (see below), these data indicate that *An. plumbeus* could play a significant role for local transmission of *P. falciparum* where there is imported malaria. In comparison, *An. labranchiae,* which is believed to have played a significant role in indigenous malaria transmission in parts of Europe, showed a lower prevalence of *P. falciparum* sporozoite infection (3 to 10%) in two independent sets of infections performed under similar conditions [23,24].

Parasite transmission depends on multiple factors which define the vectorial capacity C, i.e. the daily rate of future inoculations originating from a currently infective source, in the equation C = b m a^2^ pt / (−ln p) [46], where b is the vector competence, i.e. the proportion of vectors that develop infective parasite stages; m the vector density in relation to host density; a the vector’s daily blood-feeding rate; p the vector’s daily survival rate; t the duration of the parasite’s extrinsic incubation period in days. Thus, besides being competent to *P. falciparum*, a mosquito species needs to both sustain parasite development upon natural temperature ranges and have a daily survival rate high enough to transmit the parasite to humans.

In the experimental setting of the presented work, mosquitoes were maintained at a constant temperature of 25°C, which corresponds to the mean temperature range of July in the subtropical Mediterranean climate. Thus, the observed infectivity of *P. falciparum* can be expected to be similar under natural conditions for southern Europe. At this temperature, the extrinsic incubation period of *P. falciparum* is 12–14 days, which increases to 22–23 days at 20°C [47]. Considering the longevity of *An. plumbeus* of up to two months [42], completion of parasite development to the sporozoite stage under cooler conditions is feasible. Furthermore, recent studies indicate that, at low mean average temperatures, parasite development is faster under realistic daily fluctuating temperatures as compared to corresponding constant temperatures [48]. Altogether this suggests that *An. plumbeus* could be a competent vector for *P. falciparum* in a Central European climate.

Additional evidence of a possible role for *An. plumbeus* in the transmission of malaria comes from its ability to feed on humans (factor a), often in large numbers (factor m), due to the proliferation of this species in large man-made containers such as abandoned manure pits [16,17,25]. For example, in Alsace (north-eastern France) during the 1990’s, populations of *An. plumbeus* expanded leading to the observation of human leg landing rates of up to 365 individuals in 15 minutes (Schaffner, unpublished). At this time, the highest infestation rate was located nearby the international Basel-Mulhouse-Freiburg airport.

The source of the *An. plumbeus* used in this study was an abandoned manure pit which was discovered during mosquito field surveys in Switzerland in 2007–2010. During these surveys, *An. plumbeus* was the most frequently collected *Anopheles* species, being reported in 40 out of 396 mosquito collections (34 larval samplings; 6 adult catches/trapping of which 5 human landing catches, one BG-Sentinel™ trapping) from all urban, suburban, rural, and nature land-use zones from an altitudinal range from 268 to 858 m above sea level. In contrast, *An. claviger* s.s., *An. maculipennis* s.s. and *An. messeae* were reported at 22, 18, and three locations, respectively (Schaffner, unpublished data). Additionally, *An. plumbeus* was the third most common species after *Culex pipiens* and *Aedes japonicus* found in 3,000 vases in Swiss cemeteries, screened for invasive *Aedes* species such as *Aedes albopictus*[27].

## Conclusions

Taking into account human and parasite movements and changes in environment and climate, the data presented strongly suggest that *An. plumbeus* can play the vector role for malaria and can significantly contribute to increase the malaria transmission risk in Central-Western Europe for both *P. vivax* and *P. falciparum*, due to (1) its proven vector competence for these two parasites, (2) the occurrence of locally high population densities as a result of the recently established exploitation of man-made breeding habitats, (3) the aggressiveness of this species to humans and (4) its longevity for up to two months enabling the parasites to complete their development to the sporozoite stage. In localities showing high vulnerability for the presence of gametocyte carriers, the risk of transmission of malaria by *An. plumbeus* should be considered.

## Competing interests

The authors declare that they have no competing interests.

## Authors’ contributions

FS conceived the study and collected the wild mosquito populations. CK contributed to the wild mosquito rearing. IT and AZ performed the experimental infections. FS, CB, IT, AM and CL participated in the data analysis and interpretation and helped to draft the manuscript. All authors read and approved the final manuscript.
